# Atomic Force Microscopy micro-rheology reveals large structural inhomogeneities in single cell-nuclei

**DOI:** 10.1038/s41598-017-08517-6

**Published:** 2017-08-14

**Authors:** Michael Lherbette, Ália dos Santos, Yukti Hari-Gupta, Natalia Fili, Christopher P. Toseland, Iwan A. T. Schaap

**Affiliations:** 1 Institute of Biological Chemistry, Biophysics and Bioengineering, School of Engineering and Physical Sciences, Heriot-Watt University, Edinburgh, EH14 4AS UK; 2 School of Biosciences, University of Kent, Canterbury, CT2 7NJ UK; 3 SmarAct GmbH, D26135 Oldenburg, Germany

## Abstract

During growth, differentiation and migration of cells, the nucleus changes size and shape, while encountering forces generated by the cell itself and its environment. Although there is increasing evidence that such mechanical signals are employed to control gene expression, it remains unclear how mechanical forces are transduced through the nucleus. To this end, we have measured the compliance of nuclei by applying oscillatory strains between 1 and 700 Hz to individual nuclei of multiple mammalian cell-lines that were compressed between two plates. The quantitative response varied with more than one order of magnitude and scaled with the size of the nucleus. Surprisingly, the qualitative behaviour was conserved among different cell-lines: all nuclei showed a softer and more viscous response towards the periphery, suggesting a reduced degree of crosslinking of the chromatin. This may be an important feature to regulate transcription via mechano-transduction in this most active and dynamic region of the nucleus.

## Introduction

In eukaryotic cells, the nucleus houses the genomic material and transcription machinery that allows the cell to develop and perform its role. In different cell types, nuclei show diverse morphology and diameters that can range from 5 to 20 µm. The densely packed nucleus is the largest organelle and it is relatively rigid compared to the rest of the cell body. Therefore, its properties are a dominating feature in whole cell mechanics, not to mention a limiting factor in processes such as cell migration^1^. For example, the migration of melanoma cells was shown to be reduced by artificially stiffening their nuclei^2^. Overall, the mechanical properties of the nucleus, such as its deformability under external forces, play a key role in enabling the morphological dynamics of cells.

The mechanics of the nucleus are defined by its architecture: the exterior lamina and interior chromatin. The nuclear lamina is important in controlling the shape of the nucleus^3, 4^. Directly under the nuclear envelope, it consists of a filamentous network, several tens of nanometres thick, made up of type A (A and C) and B (B1 and B2) lamin proteins. Diseases, such as cancer, are associated with changes in the lamina composition and introduce large-scale morphological alterations to the nucleus^5^. The resulting variations in nuclei stiffness have been related to increased motility and metastatic potential^2^. A more pliable nucleus would help to squeeze through tissues during invasion, although it remains unclear if their stiffness is systematically reduced to enhance their invasive properties.

The nucleus’ interior is largely occupied by chromatin, which is comprised of DNA wrapped by histone complexes. Transcription is not uniformly distributed within the nucleus, despite the presence of the machinery throughout the organelle. It has been shown that transcription activity mostly occurs in the outer half of the nucleus, approximately from half to three-quarters of the nucleus radius^6^. Re-localisation of genes from the periphery into the nucleus interior is hypothesised to be a mechanism to activate expression^7^. The chromatin organisation within these regions is not yet completely understood but appears to rely on well-defined spatial positioning of genes and chromosome territories^8^. Historically, the chromatin is organized in heterochromatin and euchromatin, regions. Heterochromatin is most abundant at the periphery, closely associated with the nuclear lamina^9^. It was initially associated with regions of no transcription due to its high packaging density. However, it has been shown that transcription can take place in heterochromatin but is repressed by RNAi^10^. The majority of the chromatin is in the form of transcriptionally active euchromatin. However, there are still ambiguities as to how well all these regions are defined, regulated and how they change through cell signalling or stress pathways. Functional differences within the chromatin are likely a consequence of different organization details that enhance or inhibit transcription. Interestingly, because chromatin is such a closely packed polymer, any variation that affects its crosslinking will also have a large effect on its mechanical response^11^.

Forces within the cell can mechanically perturb the nucleus^12, 13^, which in turn affects gene expression^14, 15^. Here, the nucleus functions as a mechano-sensor. However, how forces are converted into deformation of the nucleus and how this leads to changes in transcription is not clear. Because lamins, whose localization is not restricted to the nuclear lamina^3^, can bind DNA, this could provide a direct mechanism for the modulation of gene expression^16, 17^. In addition, force-induced rearrangements of the chromatin^18^ itself could act as a mechanism to regulate transcription. However, as cells constantly exert forces upon the nucleus^19^, there must be significant control of this process. In other words, the nucleus must have the ability to differentiate force signals to prevent continuous aberrant changes in gene expression. One way to achieve this is a mechanical response that is sensitive to the time- and length scales of the induced deformations.

The mechanical properties of single cells and nuclei have been intensely studied. Whole cells have been shown to behave as a non-homogeneous material with a complex viscoelastic response. Cells respond stiffer when deformed at higher frequencies and it is widely agreed that their stiffness *k* obeys a weak power law: $k(f)=A{f}^{\alpha }$, where *f* is the frequency, *A* a scaling factor and *α* the exponent^20–22^. The value of the *α* lies in the range 0.1–0.35, and depends on how much the cell is deformed^23^. At deformation frequencies above ~100 Hz, the value of α increases to 0.75 and above^24–26^. This increase of *α* in a cell-like fashion can be reproduced with cross-linked networks of actin filaments^27, 28^. Modelling of such semi-flexible polymer networks identified a frequency dependence of the effective stiffness of individual polymers; because the longitudinal bending modes of single filaments need a certain time to relax, this leads to a higher effective stiffness on short time scales which results in α = 0.75^29^. In combination with cross-linking of the filaments, this can lead to the existence of various power law regimes^30^.

For the nucleus, such a consensus picture is still missing, partly because of its smaller size and also because it is difficult to separate its response from the rest of the cell, in whole-cell measurements. Nevertheless, it has become clear that both the nuclear lamina^31, 32^ and the chromatin contribute^33–35^. At different deformation length-scales, the response can be either dominated by the chromatin or lamina^36^. Measurements on nuclei lacking lamins showed that the chromatin responds nearly elastically to small (0.1 µm) deformations induced with optical tweezers^19^. At much larger deformations, induced with micropipettes, the chromatin showed a more pronounced viscous response and even plastic deformations^37^.

To quantify the visco-elastic response of isolated nuclei at a large bandwidth of length- and time-scales, we adapted an atomic force microscopy (AFM) micro-rheology method, previously developed to measure the dynamic response of whole cells^38, 39^. Individual nuclei were compressed between two plates and subjected to small oscillations at frequencies between 1 and 700 Hz. The advantage of this approach is that a single measurement can reveal the response at different time scales and that both the elastic and viscous contribution can be separated. To probe the different regions of the nucleus, the measurements were repeated at different deformation length-scales that were mapped to different nuclear regions with finite element analysis (FEA). To identify conserved features of the mechanics of nuclei, measurements were performed on four common mammalian cell lines. The insight gained in the mechanical response of the nucleus at different time- and length-scales will be helpful to interpret deformations that are observed in nuclei of migrating and differentiating cells. In addition, we have identified spatial differences of the mechanics within the nucleus and possibly in the chromatin structure, shedding light on the relation between mechanics and transcription.

## Results

### Measuring nucleus mechanics under compression between two plates

**Figure 1:**
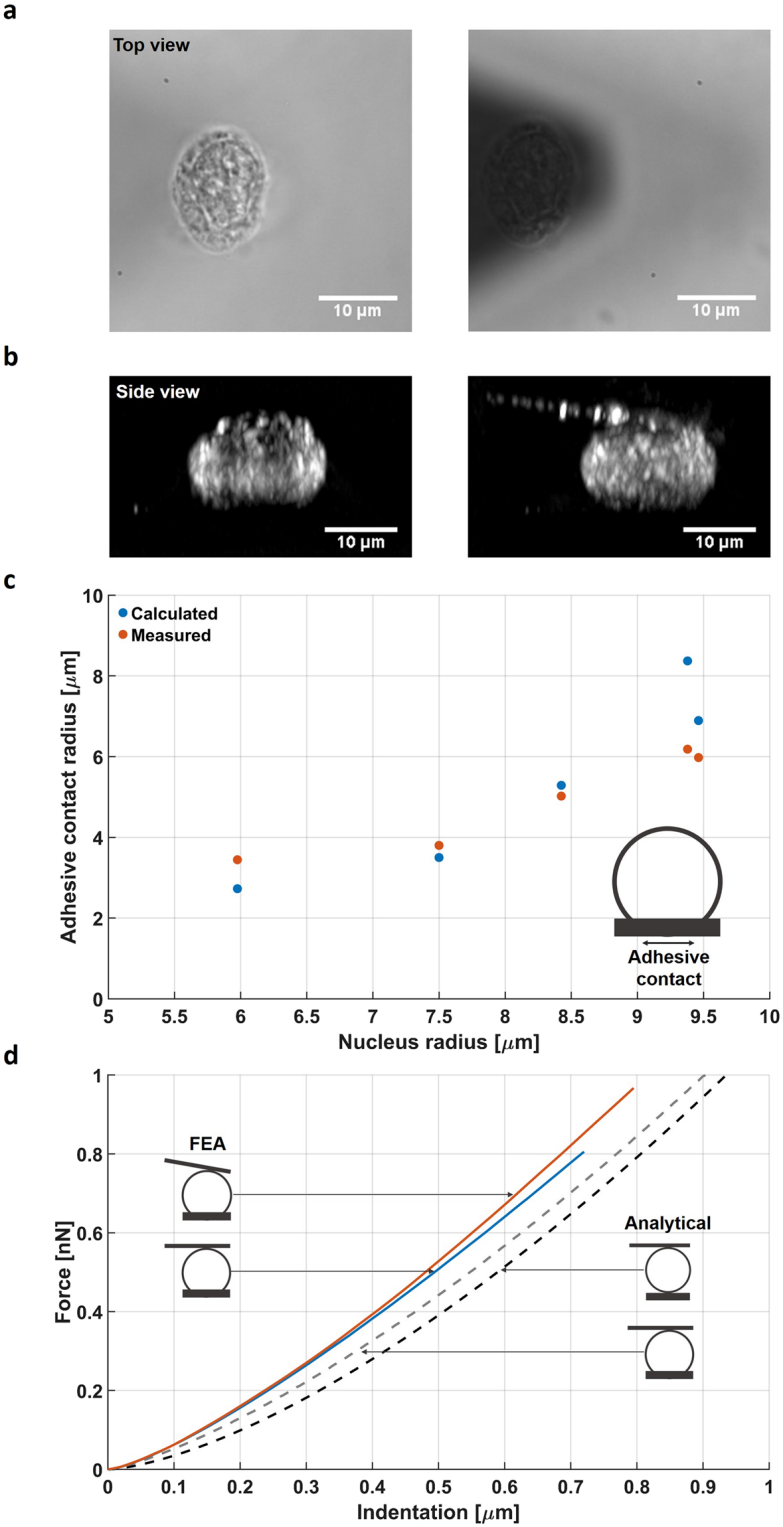
Defining contact conditions during compression of the nucleus. (**a**) Left: top-view microscopy image of the adhered nucleus. Right: the tip-less cantilever is brought down to compress the nucleus. (**b**) Left: side-view projection reconstructed from a stack of fluorescent microscopy images. The adhesive contact between nucleus and supporting surface is clearly visible. Right: the same nucleus under compression after the cantilever has been brought down. (**c**) The measured adhesive contact radii of five nuclei of different sizes (red). The calculated radii based on the height/width ratio agree within ~15% (blue). (**d**) Force versus indentation plots for comparing the calculated compression of a sphere with *E* = 2 kPa, assuming different contact conditions. Shown are two analytical solutions: the Hertzian contact (black dash line) and an extension of this model that includes the adhesive contact^42^ (grey dash line). The other two curves are from FEA and show the compression of a sphere with adhesive contact with a parallel (blue solid line) and tilted cantilever (red/orange solid line).

We used nuclei isolated from the HeLa mammalian cell line as our model system. During the measurements, single nuclei were compressed between the supporting glass coverslip on one side and the tip-less cantilever on the other. To convert the measured stiffness into a Young’s modulus that describes the intrinsic elastic properties of the nucleus, we first needed to know the contact conditions between the nucleus and both surfaces. Figure 1a,b show the microscopic top- and side-view of the nucleus, respectively, before and during the compression experiment. This was used to understand the contact boundary conditions and thus allowed us to choose which mechanical model to apply. If the nucleus behaves like a sphere compressed between planar surfaces, Hertzian contact mechanics can be used to extract the Young’s modulus. However, this commonly used approach ignores two aspects of the experimental conditions that can be seen in the microscopic images: i) the nuclei showed an initial deformation; the bottom part is flattened because of adhesion of the nucleus to the supporting surface (Fig. 1b left), and ii) the AFM cantilever is tilted 10° with respect to the supporting surface (Fig. 1b right). Both aspects are missing in the Hertz theory, but would potentially affect the calculated values.

#### Adhesive contact

We visualized the shape of five adhered nuclei, using our previously described fluorescence microscope combined with AFM^40^. The nuclear membrane was labelled and a stack of images at different focal planes was recorded with fluorescence microscopy, which allowed us to generate a 3D representation of the nucleus (Fig. 1b; see methods). This was used to measure the adhesive contact radius between the nucleus and the supporting surface. Figure 1c shows the adhesive contact radii for the five nuclei: the measured radius depends on the size of the nucleus and ranges between 3 and 6 µm. Due to the small vibrations caused by the re-focussing to record at different focal planes this method could not be applied during the mechanical measurements. Furthermore, the fluorescent labelling might affect the mechanical properties. So we tested an alternative method to obtain the adhesive contact. As shown in Fig. 1b, adhesion of the nuclei on the surface results in a decrease in height, from which in principle the adhesive contact radius can be extracted. To obtain the height reduction of each of the nuclei we combined optical microscopy for the measurement of the lateral dimensions and AFM for measuring the height. The ratio of the height and width was then used to calculate the contact radius (see methods). Figure 1c shows that the calculated adhesion radii for the five nuclei are in good agreement with the experimental values obtained from the reconstructed fluorescent cross-sections. For subsequent analysis of all nuclei (Table 1), we used the ratio of the height and width of each individual nucleus to obtain its adhesion contact radius.

**Table 1:** Dimensions of all measured nuclei. All values are mean ± s.e.m. The height was obtained with AFM. The width was obtained optical microscopy by taking the mean of the short and long axes.

	HeLa	HeLa (fresh)	IMR5	HEK	MCF7
Height [µm]	10.91 ± 0.59	16.20 ± 0.48	6.19 ± 0.35	8.31 ± 0.26	15.95 ± 0.66
Width [µm]	12.73 ± 0.54	17.18 ± 0.39	8.32 ± 0.16	10.06 ± 0.18	17.99 ± 0.51
Height/Width	0.86 ± 0.03	0.95 ± 0.03	0.77 ± 0.02	0.83 ± 0.03	0.88 ± 0.03
n	23	21	21	29	20

#### Tilt of the cantilever

The second surface contact point of the nucleus occurs at its interface with the cantilever. Because this contact occurs upon initiation of the experiment, we assumed this contact radius to increase from zero according Hertzian contact mechanics. We initially attempted to visualise this, using the reconstructed fluorescent side-view projections at different forces. However, our results were not clear because the cantilever was poorly defined and it was difficult to maintain a constant force during image acquisition (data not shown). Because the cantilever has a 10° angle with respect to the supporting surface, there will be a horizontal force component that will affect the measured stiffness. To assess the consequences, we performed FEA of the experimental geometry using a parallel or a 10° tilted cantilever (see methods). Figure 1d shows that the difference between the two cantilever geometries is relatively small (≈10%) in the simulated force versus indentation curves. This is consistent with previous experiments on whole cells in which wedged cantilevers (to achieve a 0° angle) were compared with tilted cantilevers^41^.

We compared the FEA, taking into account the adhesive contacts with the standard Hertz model. Although, this model ignores the aforementioned adhesive contact and the 10° angle of the cantilever, we found that the differences are relatively small. Recently, an analytical correction for the Hertz contact mechanics model was presented to include the adhesive contact area on spherical particles^42^, which is also plotted in Fig. 1d. Although this model still predicts a slightly softer response (≈25%) than the FEA, we chose, for the sake of reproducibility, to use this modified Hertz contact model to quantify the Young’s modulus of the following experiments. Hereafter, we will refer to the modulus as the complex Young’s modulus (E*) to indicate that this can contain both elastic (*E*′) and viscous (*E*″) components.

### The elastic modulus of the nucleus at small and large deformations

**Figure 2:**
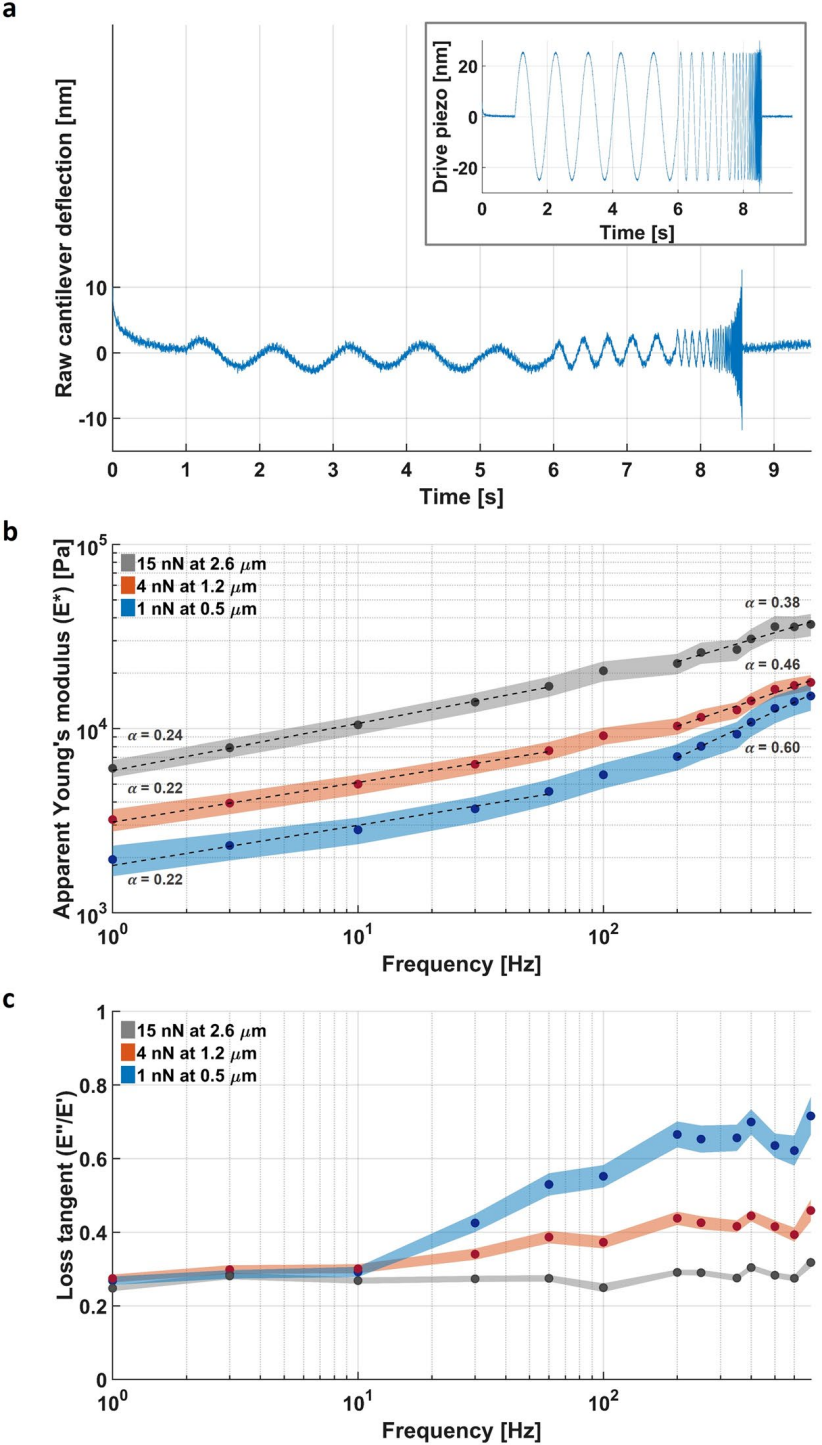
Measuring the response of the nucleus at different deformation length- and time-scales. (**a**) Inset shows the motion of the piezo-element that moves the base of the cantilever with increasing frequencies at a constant amplitude of 25 nm. The main panel shows the cantilever bending at its free end. Higher bending means more resistance, which can be induced by the compliance of the nucleus but also by the drag of the surrounding buffer. The latter is corrected for in the analysis. (**b**) At low deformation rates of 1 Hz, E* is not constant but increases threefold when the indentation depth is increased from 0.5 to 2.6 µm. At higher deformation rates up to 700 Hz, the dependency on the indentation remains. In addition, the modulus increases multi-fold with frequency. E* below and above the estimated threshold of 100 Hz had been fitted with a power law (dashed black line). The slope at low and high frequency are 0.22 and 0.60 at 1 nN, 0.22 and 0.46 at 4 nN and 0.24 and 0.38 at 15 nN. (**c**) The complex Young’s modulus can be decomposed in its viscous (*E*″) and elastic (*E*′) components. The ratio *E*″/*E*′ (loss tangent), shows that the viscous contribution is small at high deformations and low frequencies. Only at small deformations and higher frequencies does the viscosity contribution becomes more pronounced. The data is shown as the mean ± standard error of the mean of the log transformed data (shaded area).

After establishing the experimental contact conditions and the relation between indentation and E*, we performed AFM micro-rheology to measure the nucleus stiffness over a wide range of frequencies (see methods). The inset in Fig. 2a shows that, after the cantilever was brought down to compress the nucleus, it was then driven at a constant 25 nm amplitude at a frequency that increased from 1 to 700 Hz. Due to the compliance of the nucleus, the actual deflection at the end of the cantilever (Fig. 2a) will be less than the drive amplitude. The deformation and stiffness of the nucleus was calculated from the cantilever deflection (see methods), after which the aforementioned modified Hertz contact model was used to convert stiffness into E*.

First, we focused on E* at low frequency (1 Hz), which is comparable to the speed of conventional AFM indentation experiments. Compared to a whole cell, the nucleus would be expected to have a more homogeneous structure. As a consequence, the mechanical properties of an isolated nucleus would be expected to be more constant over different length scales. In the case of whole cells, which have a very inhomogeneous structure, we previously found E* to increase two-fold when the indentation increased from 0.2 to 1 µm^23^. To test this for the nuclei, we measured their mechanical response at different deformations by varying the force from 1 to 15 nN. This led to deformations between 0.5 and 2.8 µm, which corresponds to deformations between 8 and 44% of the radius, when an average nucleus radius of 6.4 µm (Table 1) is taken into account. Interestingly, as Fig. 2b shows, E* is not constant, but increases threefold at higher deformations, from 2 kPa at 0.5 µm deformation to 6.5 kPa at 2.6 µm deformation. The dependence of E* on the deformation length-scale indicates substantial spatial differences in the organization of the nucleus interior.

### The dynamic response of the nucleus between 1 and 700 Hz

The stiffness and E* of the nuclei increase as a function of the frequency (Fig. 2b). Interestingly, the mode of increase depended on the deformation length-scale. At low deformation, the slope of the curve is non-constant but increases with the frequency. At high deformation, the slope is nearly constant. Because the curves are plotted on a double logarithmic scale, a constant slope means that the curve follows a power law with a constant exponent: ${E}^{\ast }(f)=A{f}^{\alpha }$, where *f* is the frequency, *A* the modulus at 1 Hz and *α* the exponent. At high deformation, *α* increases from 0.24 at frequencies below 100 Hz to 0.38 at higher frequencies. Previous micropipette aspiration measurements, where chromatin was subjected to large multi-micrometer deformations, have shown that this chromatin power law rheology extends to much longer time-scales (100s of seconds) with *α* ≈ 0.3^33^. At low deformation, we found the increase of *α* to be larger, up to 0.6 at high frequencies. A larger *α* corresponds to a steeper curve, therefore the higher value of *α* shows that at low deformations the nucleus is more sensitive to changes in the deformation rate.

To assess the contribution of viscosity, the complex Young’s modulus was decomposed into its elastic *E*′ and viscous components *E*″ (see methods and Supplementary Fig. S1). Figure 2c shows the ratio between viscosity and elasticity, namely the loss tangent. At high deformation, the contribution of the viscosity is small (ratio of 0.3) and constant along the entire frequency range. However, at low deformation, the viscous contribution increases up to a ratio of 0.7 at higher frequencies. The differences observed in slopes and the viscous contributions measured at different deformations, further support the presence of spatial differences in the organization of the nucleus as a whole, and potentially within the chromatin.

### Modelling the spatial variation of stiffness for the nucleus

**Figure 3:**
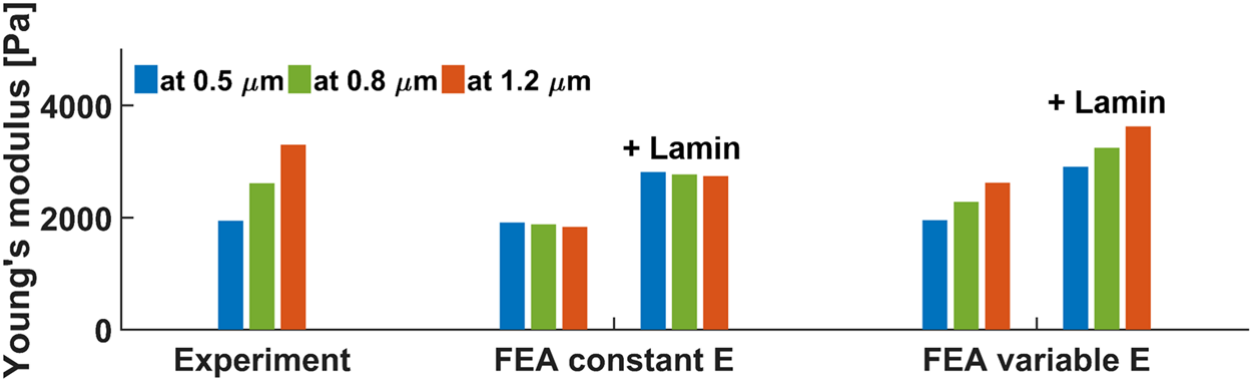
Modelling the spatial variation of the nucleus stiffness. Left: The experimental values, show a rise of E* at increasing deformation (the response at 15 nN is not shown because the resulting high deformation proved to be very difficult to simulate numerically). Middle: When the nucleus is modelled with FEA as a sphere with a constant Young’s modulus, the calculated values after analysis are nearly constant. Adding a stiff external layer, representing the nuclear lamina increases the absolute stiffness but does not introduce the deformation length-scale dependency. Right: Here, the modelled nucleus has a Young’s modulus that linearly decreased from the centre to the periphery (from 10 to 0.3 kPa). The analysed values for *E* now increase with the deformation length-scale, similar as the experimental values. This is independent of the nuclear lamina.

For small deformations, which would be limited to the outer region of the nucleus, E* is low, while at higher deformations, reaching to the interior of the nucleus, E* is much larger. To understand how the organization of the nucleus would have to vary to explain the observed response, we formulated a FEA model where *E*′ is varied as a function of the distance to the nucleus centre. Viscosity is not considered in the model. Figure 3 shows that when *E*′ is kept constant at 2 kPa, the calculated values for *E*′ (obtained by analysing the simulated stiffness curves) remain nearly identical. When *E*′ is linearly increased towards the centre, the calculated values for *E*′ increase at larger deformations. Although it is clear that the Young’s modulus increases towards the centre, a linear increase is a simple assumption and the real distribution of stiffness is likely more complicated. The presence of a stiff nuclear lamina does not affect the qualitative findings. By compressing the nucleus between two plates, we avoid localized bending of the nuclear lamina which reduces its mechanical influence in our experimental geometry. Simulations in which we included the nuclear lamina using reported elasticity values^31, 43^ show that, although the absolute stiffness increases, the dependence on the interior composition remains (Fig. 3). Remarkably, the required increase of *E*′ in the model to reproduce the experimental values is more than one order of magnitude, which is a strong indicator that chromatin, as the main component of the interior, is differently organized towards the periphery of the nucleus. The looser organization of the chromatin at the outer region results in a lower Young’s modulus and a higher viscous contribution.

### Comparing nuclei of different cell lines

**Figure 4:**
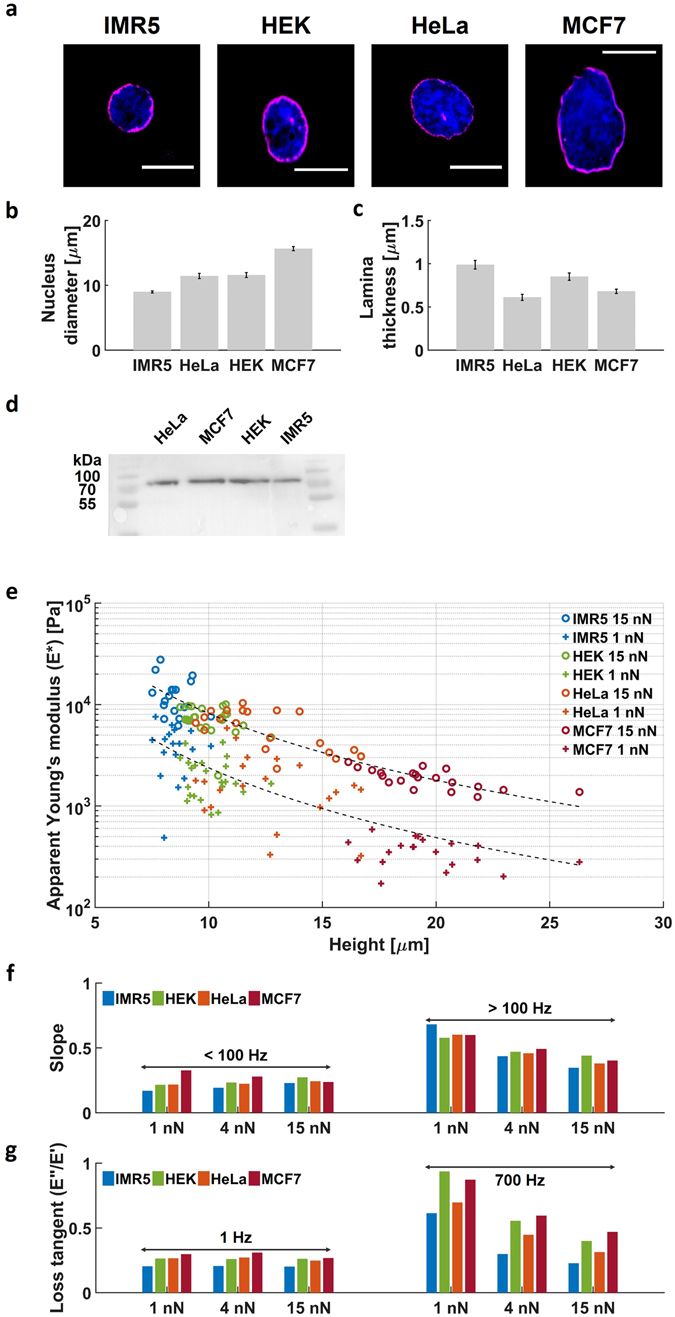
Comparing nuclei of different cell lines. (**a**) Example images from immunofluorescence staining against Lamin B with Hoechst staining for DNA in isolated IMR5, HEK293T, HeLa and MCF7 nuclei. Scale bar = 10 µm. (**b**) Diameter of the isolated nuclei determined from the immunofluorescence staining. 8.9 ± 0.2 (n = 33), 11.4 ± 0.4 µm (n = 18), 11.6 ± 0.4 µm (n = 20) and 15.6 ± 0.3 µm (n = 20), IMR5, HeLa, HEK293T and MCF7 respectively. Error bars are s.e.m. (**c**) The thickness of lamin B layer, estimated from the deconvoluted images. 0.99 ± 0.05 (n = 18), 0.62 ± 0.04 (n = 33), 0.85 ± 0.04 (n = 20) and 0.68 ± 0.02 (n = 20), IMR5, HeLa, HEK293T and MCF7 respectively. Error bars are s.e.m. (**d**) Western blot against lamin B in the nuclear extract from the cell lines used in the study. Samples were normalised to total protein content in the nuclear extract. (**e**) E* scales with the inverse of the nucleus size as shown by the fit (${E}^{\ast }\equiv {r}^{\alpha }$ with $\alpha =-2.21$ and −2.16 at 1 nN and 15 nN respectively). Nuclei appear always softer when subjected to smaller deformations. Individual responses can found in Supplementary Figure S1. The number of nuclei investigated per cell line is indicated in Table 1. (**f**) All nuclei show a slope α of the modulus vs. frequency response that only increased when probed at small deformations at high frequency. (**g**) All nuclei show a loss-tangent that only increased when probed at small deformations at high frequency.

So far we have used HeLa nuclei as our model system, however, nuclei from different cell types have diverse morphologies, which may have a large effect on their mechanical properties. Earlier work showed that all major lamin subtypes play roles in maintaining nuclear shape^4, 44^. We compared nuclei from HeLa, IMR5, HEK293T and MCF7 cells using immunofluorescence against lamin B (see methods) as a marker for the lamina. Nuclei are shown in Figure 4a and their respective size are displayed in Fig. 4b. Figure 4a shows that the smaller nucleus, IMR5, has a better-defined lamina than the largest one, MCF7. The thickness, measured from the fluorescent images, was almost double (1.0 vs. 0.65 µm; Fig. 4c). For an accurate determination of the lamina quantity, we quantified the relative expression levels by western-blot. Figure 4d essentially shows identical protein content. These observations are consistent and showed that the amount of lamin B per nucleus is almost constant but organized as a thicker layer for smaller nuclei. A thicker lamina may enable greater compression of the chromatin to generate a higher packing density, as would be required in smaller nuclei, assuming the same chromatin content between small and large nuclei. An outlier to this conclusion is the HEK293T nuclei which displayed thicker lamina than expected. However, there are likely to be cell-to-cell and cell-type variations to fit with a general trend.

Despite the difference in nuclear size, the quantity of chromatin inside, at least based on karyotype, will be approximately identical in the different mammalian cell lines tested here. Therefore, the larger MCF7 nuclei will have their chromatin less densely packed which should result in a lower Young’s modulus. To test this, we compared the stiffness of nuclei obtained from four different cell lines. Figure 4e shows that the main determinant for E* is the size of the nuclei, small nuclei are much stiffer than large nuclei. Although the packing density of chromatin has a very large effect (order of magnitude) on the absolute Young’s modulus, the difference between low and high deformations remained. This indicates that the softening towards the outside, as observed for HeLa nuclei, is preserved among all tested species and does not depend on the packing density.

This raises the question of whether the other qualitative features that we observed for HeLa nuclei are also conserved, namely the mode of increase of E* (quantified by *α* in Fig. 2b) and the increased viscous contribution at the outer region. Figure 4f shows the slopes (*α*) of the E* versus frequency plots of all different nuclei was nearly identical and unrelated to their size, with *α* always being highest at small deformation and high frequency. Also, the viscous properties are comparable for all nuclei, with viscosity always being most pronounced at small deformations (Fig. 4g). Although the isolated nuclei, that were stored at −80 °C are susceptible to buffer composition and experimental conditions, their qualitative response is remarkably conserved (Figure S2). Despite the large differences in size and absolute stiffness, all tested nuclei exhibit a comparable qualitative response, which indicates conserved features in their structural organization.

## Discussion

We have measured the visco-elastic properties of isolated mammalian nuclei by using AFM micro-rheology. By varying both the deformation length- and time-scales in combination with FEA, we could identify large spatial inhomogeneities in the structure of the nucleus. Our findings indicate there are spatial variations in the organization of the chromatin itself, rather than a homogeneous chromatin interior enveloped in a stiff lamina, which contribute to the overall mechanical properties.

All nuclei, small and large, showed a similar qualitative response. The outer region, several microns thick, is softer, more viscous and shows the strongest dependency of E* on the rate of deformation. The core of the nucleus is always stiffer and predominantly elastic. What is the cause and consequence of these two zones within the nucleus? Crosslinking has a large effect upon the mechanical response of a polymer. An increasing number of cross-links will increase the stiffness of a polymer suspension and reduce the proportional contribution of viscosity^11, 45^, which is fully consistent with our measurements. With respect to chromatin, crosslinking is achieved through condensation by condensin protein complexes and therefore, our data suggest that various levels of DNA condensation exist.

It is tempting to link crosslinking to the transcriptional activity inside the nucleus. Regions of high transcription activity have been localized in the outer half of the nucleus^6^, while also the regulation of transcription by gene repositioning takes place in the outer region^46^. Thus, the mechanically looser organization of this part of the nucleus may be required to facilitate chromatin dynamics. In the nuclear core, transcription and its regulation may be inhibited by a high degree of cross-linking, which additionally also offers an increased resistance against mechanical perturbations. The reduced stiffness of the outer region of the nucleus also provides a higher sensitivity for mechanical stimuli to facilitate mechano-sensing. Furthermore, we found the outer region to have higher sensitivity for the deformation rate: low frequencies are more easily transmitted than high frequencies. This time-scale dependent force transduction may help the nucleus to separate short-lived perturbations from consistent mechanical signals which would protect against sporadic changes in gene expression.

The absolute stiffness of the nuclei varies by an order of magnitude when compared between different cell-lines. Thus, the chromatin density, likely controlled by the nuclear lamina, appears much less critical for its proper functioning. An interesting consequence is that, as long as the nucleus maintains the aforementioned gradient of crosslinking, this may equip the cells with a mechanism to tune the size and correlated stiffness of their nuclei to match cellular function.

While isolated nuclei provide a means to precisely determine their intrinsic mechanical properties, the experimental environment is very different to the cell. However, our observation of two mechanical zones within the nucleus was independent to shrinking and swelling (Figure S2) suggesting this property is maintained. The densely packed cytoplasm exerts forces upon nuclei which may perturb both their shape and size, which would in turn change the mechanical properties. However, this is cell line dependent whereby HEK293T cells display no change in size^47^. Conversely HeLa cell nuclei have an elongated morphology of 10–20 μm with a height as long as 4 μm when grown in 2-dimensional culture. In the case of HeLa cells, the compression of the nucleus may reduce the inhomogeneity between the inner nucleus and periphery due to the reduction in volume. Intriguingly, nuclei within cells grown in 3-dimensional culture to mimic tissue grown adopt the spherical shape, as observed in the isolated nuclei. Therefore, the consistent visco-elastic properties of the several mammalian nuclei which we have observed here are likely to be conserved within the tissue microenvironment.

## Methods

### Chemicals and Reagents

Unless stated, all reagents were from Sigma Aldrich, UK.

### Nuclei isolation

HeLa, MCF-7, IMR-5 and HEK293T Cell lines were cultured at 37 °C and 5% CO_2_, in Gibco MEM Alpha medium with GlutaMAX (no nucleosides), supplemented with 10% heat-inactivated Fetal Bovine Serum (Gibco), 100 units/ml penicillin and 100 µg/ml streptomycin (Gibco).

The nuclei isolation protocol was adapted from the Collas Lab protocol^48^. Cells, plated at 90% confluency, were trypsinized in 0.05% trypsin/EDTA (Invitrogen) and harvested by centrifugation at 415 × g, at 4 °C. Cells were washed once with ice-cold phosphate-buffered saline (PBS), re-suspended in ice-cold Hypotonic Buffer (10 mM Hepes pH 7.5, 2 mM MgCl_2_, 25 mM KCl supplemented with 1 mM phenymethylsulfonyl fluoride (PMSF), 1 mM dithiothreitol (DTT) and 1x Halt Protease Inhibitor Cocktail (Thermo Fisher Scientific)) and harvested by centrifugation at 415 g, at 4 °C. Cells well then re-suspended in ice-cold hypotonic buffer and incubated for 1 hr on ice. Cells were then homogenized on ice with a glass Dounce homogeniser (Wheaton) by performing 100–150 strokes, until 90% lysis was achieved. Cell lysis was assessed on the TC20 Automated Cell Counter (Bio-Rad). Cell lysate was supplemented with 125 μl of 2 M sucrose solution per ml of lysate and mixed well by inversion. The lysate was centrifuged for at 4 °C at 184 g using a swinging bucket rotor. The pellet, which corresponded to isolated nuclei, was further cleaned by re-suspension in ice-cold Hypotonic Buffer plus 250 mM sucrose and further centrifugation at 4 °C at 184 g. The nuclei pellet was re-suspended in freezing medium (Hypotonic Buffer plus 70% glycerol) before storage at −80 °C. All measurements were performed on nuclei that were stored at −80 °C. As control we also performed measurements on nuclei that were freshly prepared. This data, included in Figure S2, shows that although the process of freezing induces shrinkage of the nuclei, the mechanical properties are fully consistent.

### AFM sample preparation

The sample was thawed and diluted 100x in Hypotonic Buffer. 100 µl was pipetted onto a 25 mm round coverslip that was mounted in the AFM coverslip holder, and left 5 minutes to adhere before the sample was washed with 500 µl Hypotonic Buffer (to remove any unfixed nuclei).

For the experiments in which the nuclear membranes were fluorescently labelled, 1 µl of a 10^4^ × diluted BODIPY® 500/510 C4, C9 stock suspension (Thermofisher; 2 mM in dimethyl sulfoxide) was added to the diluted nuclei.

### Optical microscopy

Each nucleus was imaged with the integrated inverted optical microscope. Brightfield imaging was performed to measure the lateral dimensions of the nuclei with an oil immersion objective (Nikon; 100 × 1.49 NA). Fluorescence imaging was performed with a water immersion (Nikon; 60 × 1.27 NA) to achieve a constant focus quality throughout the sample which is important to obtain a accurate 3D reconstruction. The images were recorded with an EM-CCD camera (Luca S-659, Andor technology, UK). The magnifications were calibrated with a calibration grid: 97.5 nm/px for brightfield and 166 nm/px for fluorescence.

### 3D image reconstruction from fluorescence microscopy images

To measure the adhesive contact radius and initial deformation of the nucleus we performed z-stacks through adhered HeLa nuclei that were fluorescently labelled. The sample was excited with a 488 nm laser and a stack of 150 images spaced by 166 nm was recorded for each nucleus. A custom written routine in LabVIEW (National Instruments, TX, USA) was used to control the objective scanner (PIFOC, Physik Instrumente, Germany) and to trigger frame acquisition on the camera (50 ms exposure time).

The z-stacks were post-processed using *ImageJ* and the *DeconvolutionLab* plugin^49^ to perform a 3D deconvolution. The point spread function was calculated using the Born and Wolf model^50^. For the figures we used side-view projections through deconvolved z-stacks using the maximum intensity values.

The adhesion contact radius was quantified by manually plotting a circle around the nucleus to get the intersection with the supporting surface.

### Immunofluorescence and imaging

Purified defrosted nuclei were immobilised on Poly-D-lysine (MW 70,000–150,000, Sigma) coated 13 mm glass coverslips (ThermoFisher). Immobilised nuclei were stained for 10 min at 37 °C with 1 μg/ml Hoechst 33342 (ThermoFisher) in Hypotonic Buffer. Stained nuclei were fixed in 4% (w/v) paraformaldehyde (PFA) and residual PFA was quenched with 50 mM ammonium chloride. Nuclei were permeabilised and simultaneously blocked with 0.1% (v/v) Triton X-100 and 2% (w/v) BSA in Tris-buffered saline (TBS). Nuclei were then immunostained against the endogenous lamin B with the rabbit anti-Lamin B1 polyclonal antibody (Abcam) and subsequently the donkey anti-rabbit Alexa Fluor 555-conjugated antibody (1:500, Abcam), both diluted in 2% (w/v) BSA in TBS. Coverslips were mounted on microscope slides with Mowiol (10% (w/v) Mowiol 4–88, 25% (w/v) glycerol, 0.2 M Tris-HCl, pH 8.5), supplemented with 2.5% (w/v) of the anti-fading reagent DABCO (Sigma).

Nuclei were visualised using Olympus IX71 microscope with PlanApo 100xOTIRFM-SP 1.49 NA objective mounted on a PIFOC z-axis focus drive (Physik Instrumente, Karlsruhe, Germany), and illuminated with an automated 300W Xenon light source (Sutter, Novato, CA) with appropriate filters (Chroma, Bellows Falls, VT). Images were acquired using a QuantEM (Photometrics) EMCCD camera, controlled by the Metamorph software (Molecular Devices). The whole volume of the nuclei was imaged by acquiring z-sections with a spacing of 200 nm. Images presented here correspond to a middle section of the nucleus. Images were deconvolved with the Autoquant X software applying blind deconvolution and analysed by ImageJ. The nuclei diameter and lamina thickness was calculated by plotting *x* and *y* intensity profiles across the nuclei. Point-to-point distances were then measured across the peaks and nuclear body. The quoted values were calculated from the average of the *x* and *y* values.

### Immunoblot analysis

Nuclei lysates were prepared by direct lysis of 4 × 10^6^ freshly defrosted nuclei in NuPAGE sample buffer, followed by 5 minutes sonication. The total protein concentration of the nuclei fraction was determined by Bradford Assay (Sigma) following the manufacturer’s instructions. Nuclei were heat-denaturated and resolved by SDS-PAGE on an 8% acrylamide gel. Proteins were transferred to 0.45 µm PVDF membrane using semi dry Power Blot Cassette (Thermo scientific). The membrane was blocked for 2 hours at room temperature with 5% (w/v) skimmed dried milk, 0.1% (v/v) TWEEN-20 in TBS and then probed against lamin B by incubation with the rabbit anti-Lamin B1 polyclonal antibody (Abcam) and subsequently a goat anti-rabbit antibody coupled to horseradish peroxidase (Abcam). The bands were visualised using the ECL Western Blotting Detection Reagents (Invitrogen) and the images were taken using Syngene GBox system. Images were processed in ImageJ.

### Contact mechanics model

For a sphere that gets compressed between two planes, the indentation (*δ*) as function of the force (*F*) is given by the double contact Hertz model in eq. 1 ^42^. *R*_*n*_ is radius of the nucleus, *E* the Young’s modulus and *v* the Poisson ratio which was set at 0.4.

1 $$\delta (F)=2{(\frac{3F(1-{\nu }^{2})}{4E\sqrt{{R}_{n}}})}^{\frac{2}{3}}$$

To include the adhesive contact radius between the nucleus and the supporting surface as seen in the experiments we used a modified Hertz contact model as derived by Glaubitz *et al*.^42^. Eq. 2 inludes the surface adhesion energy *γ*, which is a function of the adhesive contact radius (*Rc;* eq. 3).

2 $$\begin{array}{rcl}\delta (F) & = & {(\frac{3F(1-{\nu }^{2})}{4E\sqrt{{R}_{n}}})}^{\frac{2}{3}}\\  &  & +{(\frac{3(1-{\nu }^{2})(F+6\gamma \pi {R}_{n}+\sqrt{12\gamma \pi {R}_{n}F+{(6\gamma \pi {R}_{n})}^{2}})}{(4E\sqrt{{R}_{n}})})}^{\frac{2}{3}}\\  &  & -{(\frac{9\gamma \pi (1-{\nu }^{2})}{E})}^{\frac{2}{3}}{{R}_{n}}^{\frac{1}{3}}\end{array}$$

3 $$\gamma ({R}_{c})=\frac{{{R}_{c}}^{3}E}{{{R}_{n}}^{2}\pi 9(1-{\nu }^{2})}$$

The adhesive contact radius can be calculated according to Hertzian contact mechanics via eq. 4. *R*_*n*_ is defined as half the diameter as measured in the optical microscopy image of the nucleus. *b* is the initial deformation induced by adhesion, defined as *b* = 2*R*_*n*_ − *height*, where the height of the nucleus was measured with AFM as described in the next section.

4 $${R}_{c}(b)=\sqrt{{R}_{n}b}$$

### Atomic force microscopy

All experiments were performed on an MFP-3D AFM (Asylum Research, CA, USA) that was mounted on a custom made inverted optical microscope which is described in detail in ref. 40. For all measurements we used tip-less MLCT-O10 cantilevers (Bruker, CA, USA) that have a triangular geometry of 170 µm long, a width of 22 µm width per arm and a resonance frequency around 22 kHz. The spring constant of each cantilever was calibrated with the built-in calibration routine based on the thermal noise spectrum and the equipartition theorem. The average spring constant was *k* = 0.069 ± 0.003 N/m (n = 11).

### AFM micro-rheology

Prior to each mechanical measurement, the dimensions of the selected nucleus were measured. First, a bright field image was recorded and the length of long and short axes were averaged to get the mean width. To obtain the height we used the AFM. By performing a force curve on top of the nucleus and one next to it on the substrate, we could extract the difference in piezo extension that was required to establish contact which gives the (undeformed) height of the nucleus.

For the measurement, the cantilever was brought down onto the nucleus at a pre-set force to achieve the desired indentation. 1 second after contact, the cantilever was oscillated at 25 nm drive amplitude at its basis, at a frequency that increased from 1 to 700 Hz in the course of 7.5 s, after which the cantilever was retracted. Because the response of the Asylum MFP3D z-scanner is only linear up to ~100 Hz the the amplitude of the drive signal was gradually increased at higher frequencies to maintain a constant oscillation amplitude over the whole bandwidth.

For the analysis, the recorded cantilever deflection curves must be converted into the stiffness of the nucleus at each frequency. This stiffness, which depends on both the visco-elastic properties and the contact mechanics of the nucleus, is eventually used with eqs 2 and 3 to extract E*. However, the drag of the surrounding liquid leads an additional increase of the measured cantilever deflection when it is moved. This is especially pronounced at high frequencies and needs to be corrected for in the calibration procedure.

In order to exclude that the response at higher frequencies is biased by changes to the material induced by the preceding measurements at lower frequencies, we performed successive control experiments at identical measurement conditions on the same nucleus. Supplementary Figure S3 shows that there is no systematic change between the measured responses, which confirms that the dynamic oscillations during the first measurement do not bias the results of the second measurement.

#### Stiffness of the nucleus

Basically, the stiffness of the nuclei follows the ratio between the applied force and the sample deformation. Although we perform cyclic deformation experiments, this method is conceptually the same as performing nano indentation experiments on the sample. The stiffness of the nucleus is obtained by dividing the force amplitude of the oscillation signal by the deformation amplitude of the nucleus (eq. 5). The force amplitude is obtained by multiplying the cantilever deflection signal (*d*) with the cantilever spring constant *k*. The deformation amplitude is obtained from subtracting the cantilever deflection from the piezo drive signal (*z*).

All analysis was performed with MATLAB (Mathworks, MA, USA). The data was analysed in frequency domain by applying a rectangular-windowing discrete Fourier analysis that allows us to extract the amplitude and phase of each signal and calculate the apparent stiffness (*K*) of the nucleus. *f*_*i*_ and the absolute values of *d(f*_*i*_) and *z(f*_*i*_) are the frequency and amplitudes of oscillation, respectively.

5 $$K({f}_{i})=\frac{k|d({f}_{i})|}{|z({f}_{i})|-|d({f}_{i})|}$$

In addition, the phase shift (*P*_*s*_) between the piezo drive signal and the cantilever deflection was calculated (eq. 6), which is used to separate the elastic and viscous components of the measured response. *P* is the phase. The argument (arg), denotes the phase of each signal.

6 $$Ps({f}_{i})\,=\,{\rm{\arg }}(d({f}_{i}))\,-\,{\rm{\arg }}(z({f}_{i}))$$

#### Cantilever drag correction

The calculated stiffness need first to be corrected for the viscous drag of the buffer that acts on the cantilever. We followed the procedure described by Alcaraz (equation 4 in ref. 51) to subtract the dynamic response of the environment and isolate the one of the nucleus. Briefly, the experiments were repeated at known distances away from the substrate. The measured cantilever deflection and phase shift will now only result from the hydrodynamic drag and the instrument response and can be parametrized at each frequency with respect to the distance from the substrate. When a measurement is performed on a nucleus, these values are subtracted from the cantilever deflection signal taking the actual height of the cantilever above the substrate into account. Values of the drag coefficients as function of the distance between the tip-less cantilever and substrate are shown in Supplementary Figure S4.

#### Calculating E*, E′ and E″

The nucleus stiffness includes both the viscous and elastic response in an unknown ratio (the additional drag on the cantilever from the surrounding liquid is calibrated and subtracted). Via a contact mechanics model first the apparent Young’s modulus (E*) is calculated. Assuming the contact area to remain constant at the small oscillation amplitude, we can use the stiffness of the nucleus to calculate its viscoelastic properties. First, the contact radius is calculated based on the diameter and indentation of the nucleus with eq. 4. Then, after applying the cantilever drag correction, we obtain the nucleus’ force and indentation amplitudes during the oscillatory stimuli. Finally, E* is calculated incorporating eq. 3 into eq. 2, which is numerically solved. E* will still be composed of a viscous and elastic contribution in an unknown ratio. To separate E* in its elastic (E′) and viscous components (E″) we use the phase shift (Ps) between the drive and response signal. If the signals are in phase the response is purely elastic, if they are out of phase by ½π, the response is purely viscous. E* can thus be decomposed by $E^{\prime} ({f}_{i})={E}^{\ast }({f}_{i})cos(Ps({f}_{i}))$ and $E^{\prime\prime} ({f}_{i})={E}^{\ast }({f}_{i})sin(Ps({f}_{i}))$. Finally, the loss tangent is defined as the ratio between viscous and elastic component, (eq. 7).

7 $$Loss\,Tangent({f}_{i})=\frac{{E}^{^{\prime} }({f}_{i})}{{E}^{^{\prime\prime} }({f}_{i})}={\rm{t}}{\rm{a}}{\rm{n}}({{\rm{P}}}_{{{\rm{s}}}_{{\rm{corrected}}}}({{\rm{f}}}_{{\rm{i}}}))$$

#### Fitting of E* with a power law

We fitted ${E}^{\ast }$ below 100 Hz and above 100 Hz, using a power law of the form ${E}^{\ast }(f)=A{f}^{\alpha }$, where *A* is a scaling factor, α is the exponent and *f* the frequency. These coefficients are estimated using a non-linear regression method. In cell mechanics a transition around 100 Hz is often reported^24, 25^ and also in our data the transition is most pronounced at this frequency. Fitting with a double power law did not allow us to clearly separate the low frequency response form the high frequency response, which is due to our still rather limited bandwidth and the fact that in some case the difference between the exponents of the power law is very small which makes that the transition region is less well defined^22^.

### Finite element analysis of nucleus deformation

All models were built with Comsol Multiphysic 5.2a (Comsol, Sweden) The nucleus was described as a sphere with a radius of 5.5 µm resting on a planar surface. The Young’s modulus was set to 2 kPa to best match our experiments and the Poisson ratio was set to 0.4.

To mimic the adhesion and initial deformation, a boundary force over the lower boundary of the sphere was applied to pull the sphere closer to the substrate. This force was adjusted to reach an initial contact radius of 1.75 µm, consistent with the one measured from optical microscopy results. Finally, the tip-less cantilever, described by a plate tilted by 10° with respect to the substrate, was lowered to indent the sphere. Contacts between the substrate, sphere and nucleus, were described with contact pairs and the contact-penalty methods according to the manufacturer’s instructions.

We used a 3D model to investigate the effect of the tilted cantilever on the forces measured. For the measurements in which we varied the Young’s modulus in the sphere we used a 2D axisymmetric model to reduce the computation time. In these models the cantilever was not tilted but parallel to the substrate.

The 2D model was also used to investigate the effect of a non-constant Young’s modulus. For these models the initial deformation was not included. The Young’s modulus was described by a linear decrease of the Young’s modulus from 10 kPa at the centre to 0.3 kPa at the periphery. This is the simplest assumption which can explain the experimental behaviour seen at different indentation depth. The lamin layer was described a 100 nm thick layer with a Young’s modulus of 200 kPa which results in an elastic area compressibility modulus of 24 mN/m, similar to value reported by Dahl *et al*.^31^. Because the thickness of the actual lamina is very thin with respect to the nucleus radius its response will be dominated by in-plane stretching and not by bending. Thus, the used thickness (*t*) of the layer in our model is not critical as long as the elastic area compressibility modulus (a product of *E* and *t*) is correct.

## Electronic supplementary material

Supplementary Information
