# Supplementary material for: Atomic Force Microscopy micro-rheology reveals large structural inhomogeneities in single cell-nuclei
Source: Sci Rep. 2017 Aug 14;7:8116. doi: 10.1038/s41598-017-08517-6 (PMC5556037; doi:10.1038/s41598-017-08517-6)
Supplement: Supplementary file 1 — Supplementary Information [file 41598_2017_8517_MOESM1_ESM.pdf]

Supplementary Information

Atomic Force Microscopy micro-rheology reveals large structural inhomogeneities in single cell-nuclei

Michael Lherbette[1#], Ália dos Santos[2#], Yukti Hari-Gupta[2], Natalia Fili[2], Christopher P. Toseland[2$*], Iwan A.T. Schaap[1$]

[1] Institute of Biological Chemistry, Biophysics and Bioengineering
School of Engineering and Physical Sciences
Heriot-Watt University
Edinburgh EH14 4AS, UK

[2] School of Biosciences,
University of Kent,
Canterbury CT2 7NJ, UK

[#] Equal contribution
[$] Joint corresponding author
[*] Address correspondence to: c.toseland@kent.ac.uk

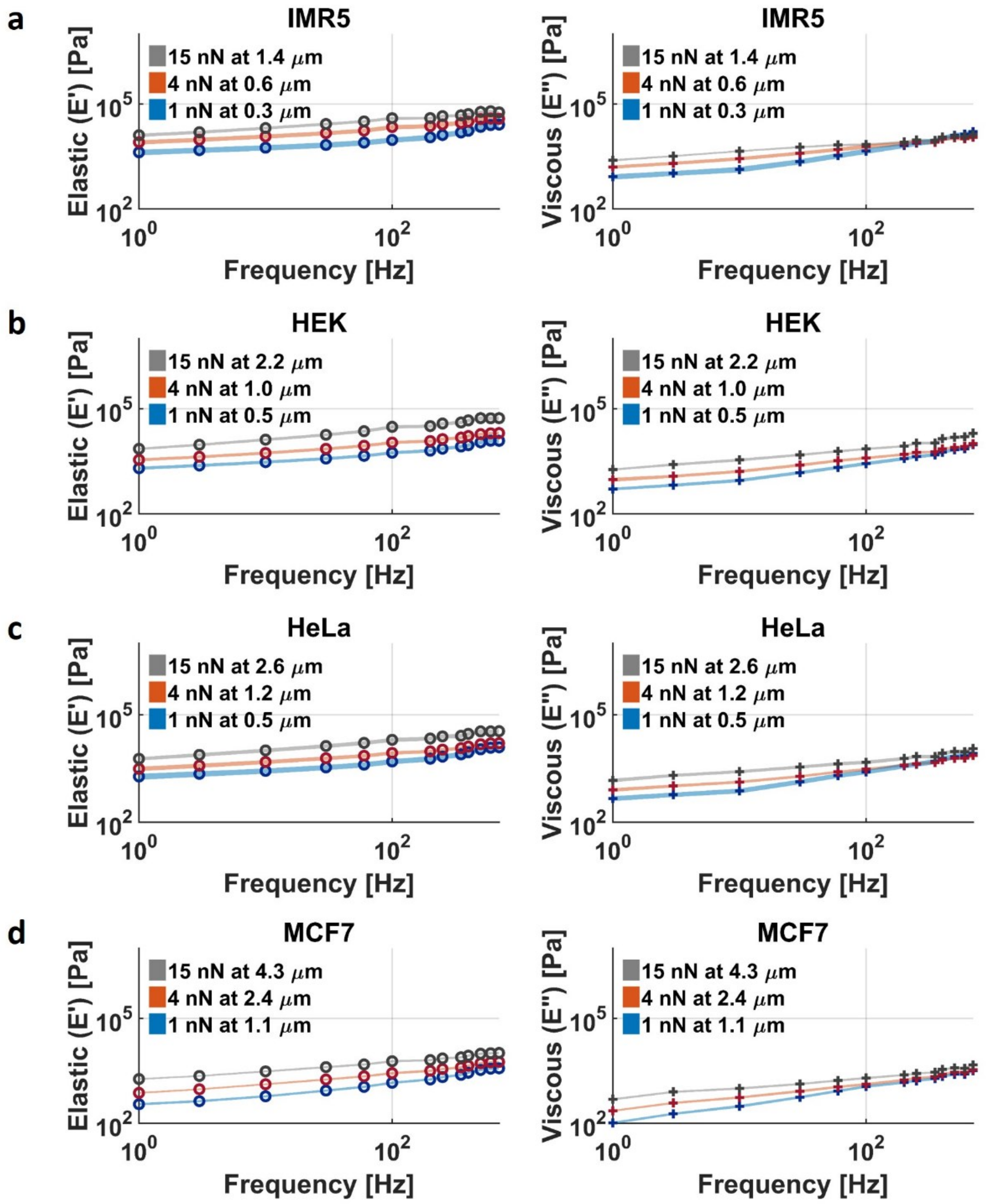

Figure S1. The elastic (E’) and viscous (E”) response of the nuclei from different cell lines. The graphs are sorted by the average size of the nuclei, from small to large.
a) IMR5, b) HEK, c) HeLa, and d) MCF7.

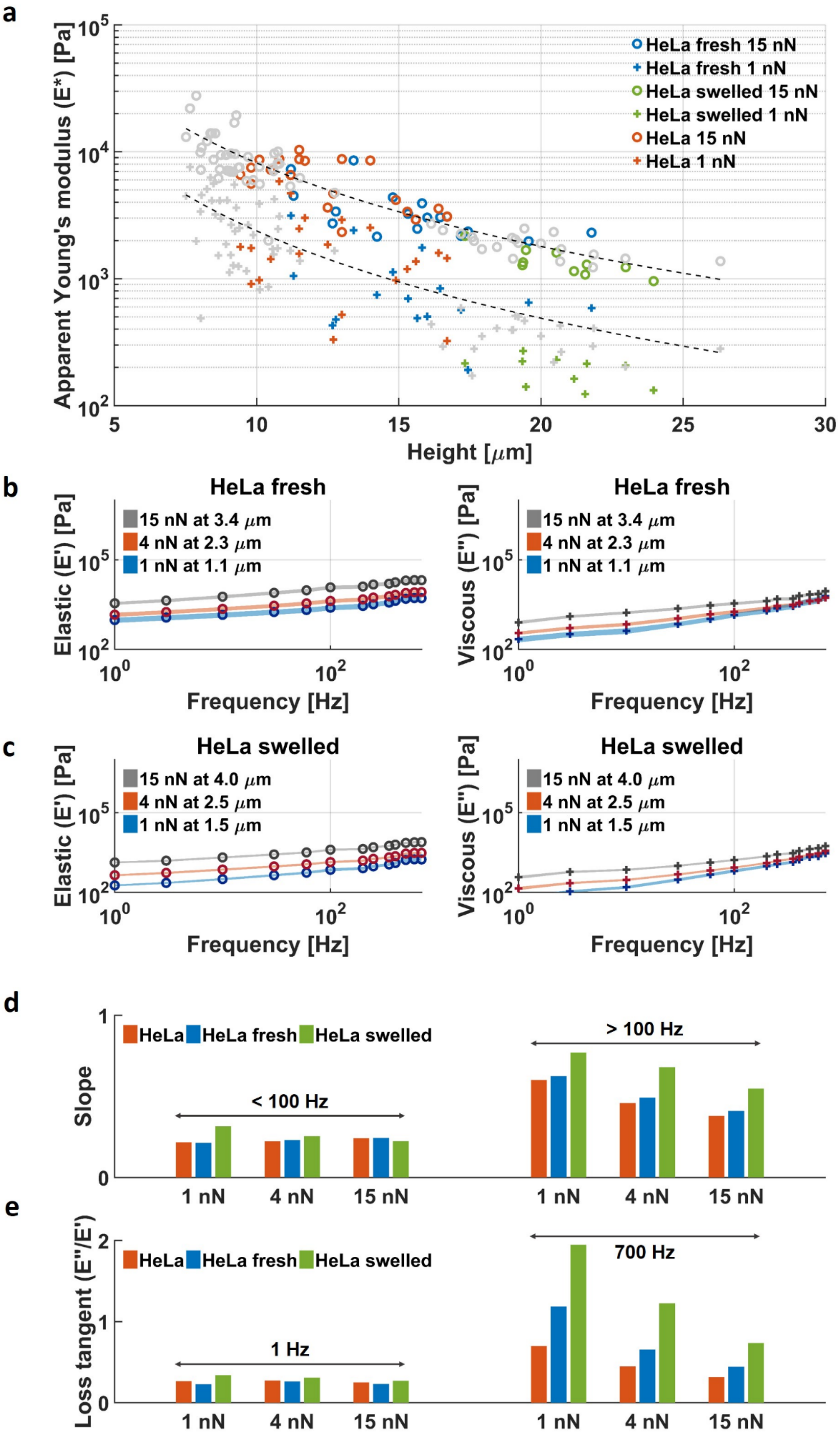

a
Apparent Young's modulus (E*) [Pa]
Height [μm]
HeLa fresh 15 nN
HeLa fresh 1 nN
HeLa swelled 15 nN
HeLa swelled 1 nN
HeLa 15 nN
HeLa 1 nN
b
HeLa fresh
Elastic (E') [Pa]
Viscous (E'') [Pa]
15 nN at 3.4 μm
4 nN at 2.3 μm
1 nN at 1.1 μm
Frequency [Hz]
c
HeLa swelled
Elastic (E') [Pa]
Viscous (E'') [Pa]
15 nN at 4.0 μm
4 nN at 2.5 μm
1 nN at 1.5 μm
Frequency [Hz]
d
Slope
HeLa
HeLa fresh
HeLa swelled
< 100 Hz
> 100 Hz
1 nN
4 nN
15 nN
e
Loss tangent (E''/E')
1 Hz
700 Hz

Figure S2. Effect of the storage conditions and the artificially swelling of HeLa nuclei.
'HeLa' nuclei' are the ones presented in the main paper and have been stored at -80 °C after isolation. HeLa fresh' nuclei were directly investigated after isolation to avoid storage at -80 °C. 'HeLa swelled' were HeLa nuclei, previously stored at-80 °C, which were subjected to hypotonic condition to induce osmotic swelling a) The response of 'HeLa' (orange), 'HeLa fresh' (blue) and 'HeLa swelled' (in green) reveal a size-dependent behavior similar to that of the nuclei of the different cell lines, the larger, the softer. To facilitate the comparison, the results from the IMR5, HEK and MCF7 nuclei are added in grey. The 'HeLa fresh' nuclei are larger than the 'HeLa' nuclei (17.2 ± 0.39 µm (n = 16) vs. 12.73 ± 0.54 µm (n = 23)), whereas, the swelled nuclei were even larger (20.7 ± 0.61 µm (n = 10)). b) Frequency dependent E*response of the 'HeLa fresh' nuclei and c) 'HeLa swelled' nuclei at different indentation depth. D) Values of the slope below and above 100 Hz are shown in. At low frequencies, the difference between differently treated HeLa samples is very small. However, at high frequencies, the swelled nuclei have the highest slope e) Differences between the loss tangent are noticeable at high frequencies. Notably, the response of the 'HeLa swelled' is clearly dominated by viscosity,

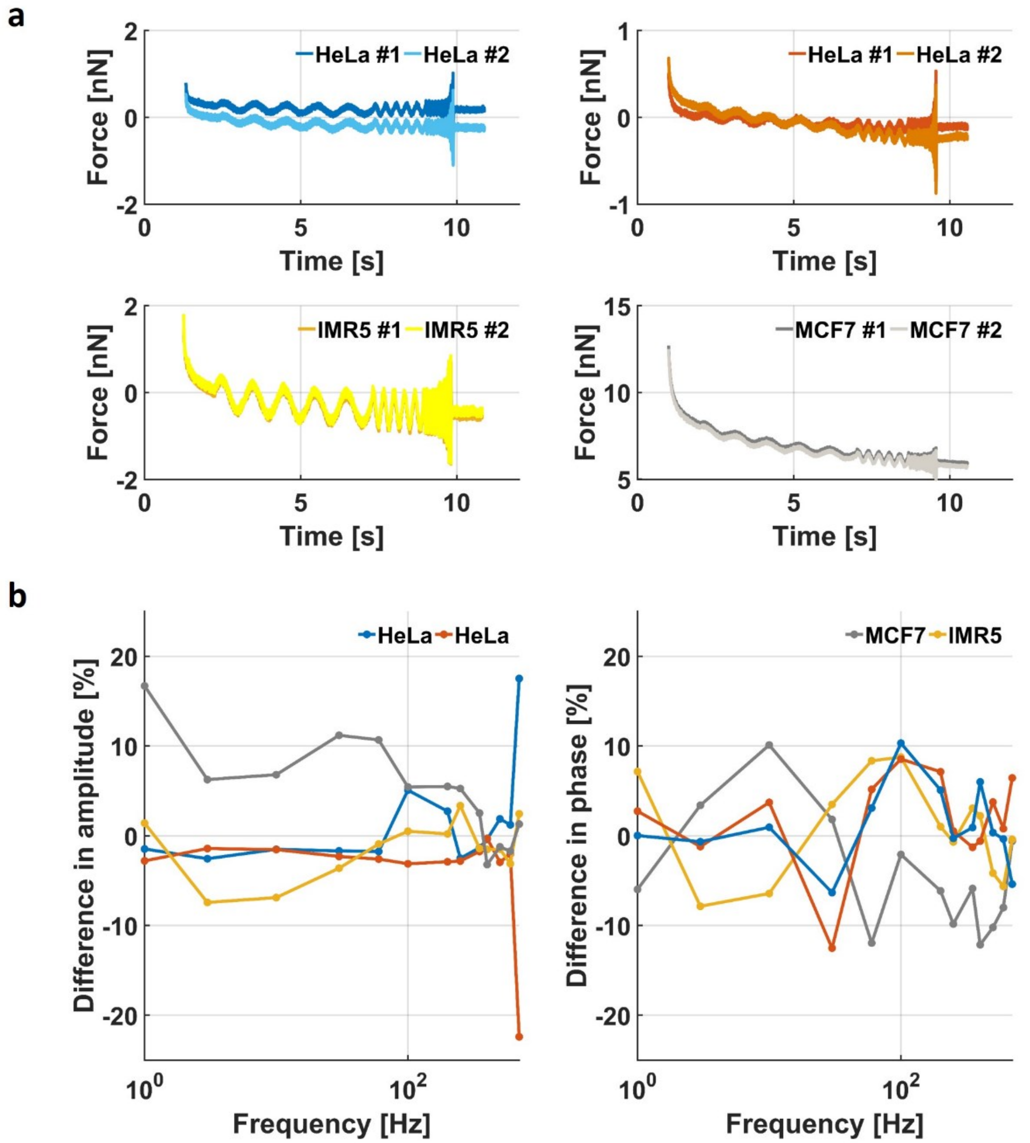

Figure S3. Repetitive experiments performed on the same nucleus show an identical response.
a) Raw data curves for 4 different nuclei from three different cell lines, HeLa (blue and red), IMR5 (yellow) and MCF7 (grey). The dark curves represent the first frequency sequence and the light curves the second. The slight differences in force originate from the thermal drift that is present during the AFM measurements.
b) The differences between the successive experiments of the amplitude and of the phase with respect to the drive signal. The differences are mostly below 10 % and do not show a systematic trend. This confirms that the first oscillation experiment does not bias the outcome of the second oscillation experiment.

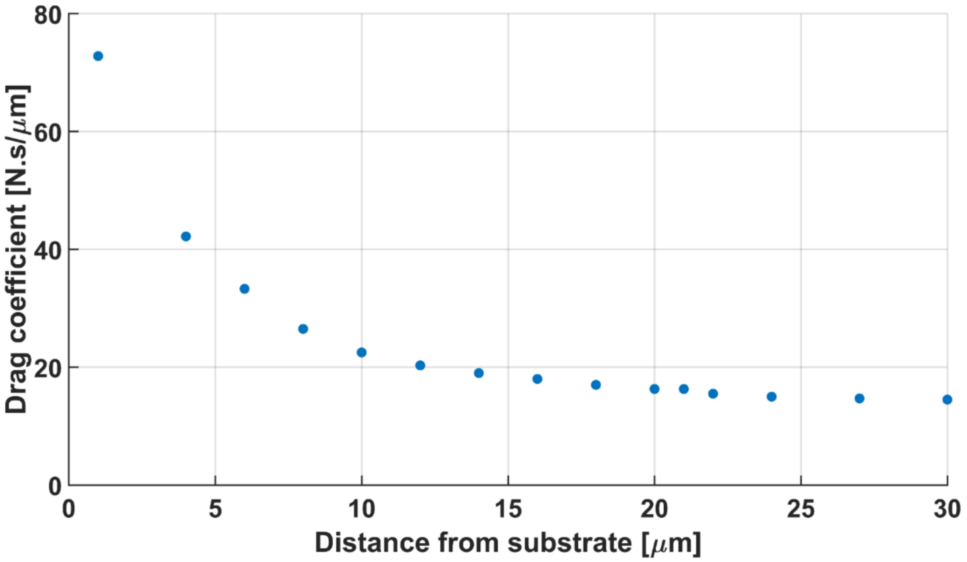

Figure S4. Drag coefficient of the cantilever as function of the cantilever-substrate separation. Drag coefficients were obtain by performing the oscillation experiment at increasing cantilever-substrate separations. Measurements were performed in the buffer used for the nuclei experiments. The drag coefficient decreases when the cantilever is further from the substrate. During the actual experiments on nuclei the cantilever-substrate separation is dictated by the height of the nuclei, typically between 10 and 20 μm.

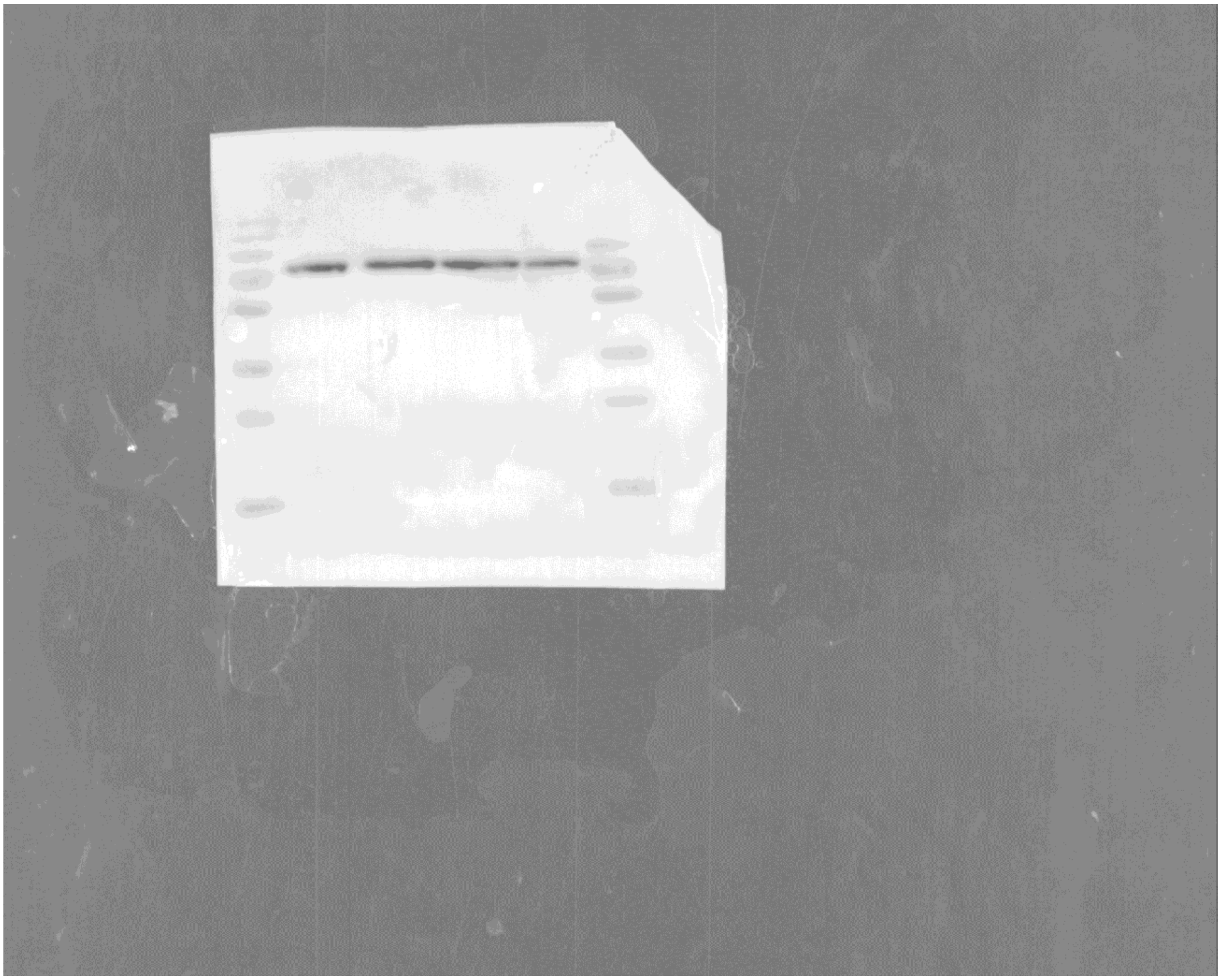

Figure S5. Full size blot from Figure 4d.
